# The application of thermal conductivity measurements to the Kuqa River profile, China, and implications for petrochemical generation

**DOI:** 10.1186/2193-1801-2-580

**Published:** 2013-10-30

**Authors:** Jiarui Feng, Zhiyong Gao, Rukai Zhu, Zhong Luo, Linyan Zhang

**Affiliations:** Central Laboratory of Geological Sciences, Research Institute of Petroleum Exploration Development, Beijing, 100083 China; Institute of Geomechanics, Chinese Academy of Geological Sciences, Beijing, 100081 China

**Keywords:** Thermal conductivity, Gypsum-bearing rocks, Kuqa River, Hot disk, Petrochemical generation

## Abstract

Measurement of thermal conductivity of rocks is important to understand the thermal properties of earth materials, the characteristics of terrestrial heat flow, and the formation of oil. In this paper we report thermal conductivity, thermal diffusivity, and heat capacity data for 12 conglomerate, sandstone, and gypsum-bearing samples from the Paleogene Kuqa River profile in Kuqa, China. Samples were measured via the hot disk technique, yielding thermal conductivity values of 0.436 to 0.998 W/mK, thermal diffusivity measurements of 0.395 to 1.314 mm^2^/s, and heat capacity values of 0.439 to 1.717 MJ/m^3^K. These analyses reveal that gypsum-bearing rocks, with their low thermal conductivity, can act as excellent insulators over oil and gas reservoirs, aiding the formation and thermal maturation of petroleum.

## Background

Thermal conductivity (*k*) is the heat conduction capacity of rock, and is defined as the direct heat conduction of the rock per unit area and unit length, under unit temperature difference and unit time. Thermal conductivity is an important parameter that controls the thermal properties of a rock, the paleotemperature evolution of a region, as well as the thermal state in the deep Earth. Thermal conductivity measurements of rocks also have important implications for oil and gas exploration, via understanding the distribution of terrestrial heat flow and evaluating the thermal evolution of oil-bearing basins. Research of rock thermal conductivity has therefore attracted more attention in recent years, as a result of an increase in petroleum exploration and research on petroleum formation mechanisms.

Based on reports of thermal conductivity for different rock types (mainly sedimentary), scholars have focused on how temperature controls thermal conductivity (Korte and Brouwers 2010; Zoth et al. 1988; Seipold 1998). For example, using measurements of rock thermal conductivity in the Tarim Basin, Wang et al. (1995a) determined the distribution of terrestrial heat flow, and found that the basin center had the highest heat flow, and lower heat flow in the depression area. Using data for thermal conductivity, heat flow, and radioactive heat generation in the Qaidam Basin, Qin (2001) calculated the temperature at depth, and found low temperatures in the northeast and high values in the central west of the basin. For the Subei Basin, Song et al. (2011) statistically analyzed the thermal conductivity of drill core samples, and found that the thermal conductivity of the Paleogene–Upper Cretaceous rocks is closely related to lithology, with the thermal conductivity of sandstone usually being higher than that of shale. Despite these extensive studies of the thermal conductivity of rocks in oil basins, there is a notable gap in the data, with few measurements on gypsum-bearing evaporites.

Evaporites are common rocks in oil reservoirs around the world, and the formation of many oil and gas reservoirs is generally related to evaporites. Major minerals in evaporites are gypsum and anhydrite, with minor amounts of halite, other mineral salts, dolomite, clay, organic matter, and iron oxides (Shen et al. 2001). Because of the physical characteristics of gypsum-bearing evaporates, especially their low permeability and porosity, gypsum-bearing rocks can act as high-quality cap rocks or reservoir beds within oil deposits (Wang et al. 2005; Gong and Zeng 2003; Ma et al. 2000; Zhang and Tian 1998; Jowett et al. 1994; Kirkland and Evans 1980). Despite this importance of gypsum-bearing rocks in oil reservoirs, there are few reports of the thermal conductivity of gypsum. To explore the thermal properties of gypsum and their impact on hydrocarbon generation, we measured the thermal conductivity of 12 rocks, including 6 rocks containing gypsum. Our study focuses on the Paleogene Kuqa River profile, in the Kumugeliemu Group, from the northeastern part of the Kuqa depression, China.

## Results and discussion

### Geological setting

**Figure 1:**
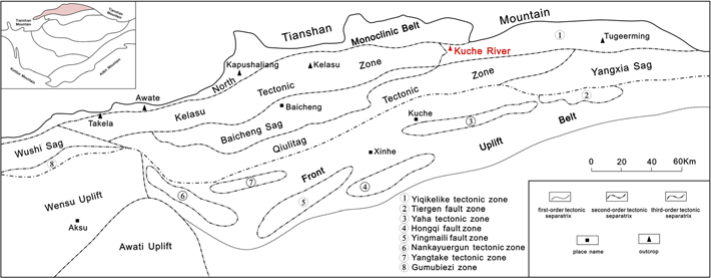
**The structure distribution map of North Tarim.** (According to the Institute of Tarim Oilfield, 2005, modified).

The Kuqa depression is located in the north of the Tarim Basin, adjacent to the Tianshan fold belt, and is an approximately east–west trending foreland basin that is dominated by Mesozoic and Cenozoic sediments (Zhu et al. 2009). This region has experienced multi-phase tectonic activity that has produced four tectonic belts (the northern monocline belt, the Kelasu–Yiqikelike structural belt, the Qiulitage structural belt, and the Tabei uplift belt) and three depressions (the Baicheng, Yangxia, and Wushi depressions) (Figure 1).

Discordantly overlying the top of the Cretaceous Bashijiqike Formation are Paleogene gypsum-bearing clastic rocks that include fossils of ostracods, charophytes, pollen, and gastropods. These Paleogene units of the Kuqa depression crop out widely in the region, with greatly varying lithology. There are obvious lithological differences between the central and marginal parts of depression, and between west and east areas (Zhu et al. 2009). The Paleogene lithostratigraphy is divided into the Talake, Xiaokuzibai, and Suweiyi formations. Rocks that cannot be easily classified into Talake and Xiaokuzibai formations are collectively referred to as the Kumugeliemu Group (E_1–2_km) (E-Paleogene; 1-2-the first and second formations; km-Kumugeliemu).

**Figure 2:**
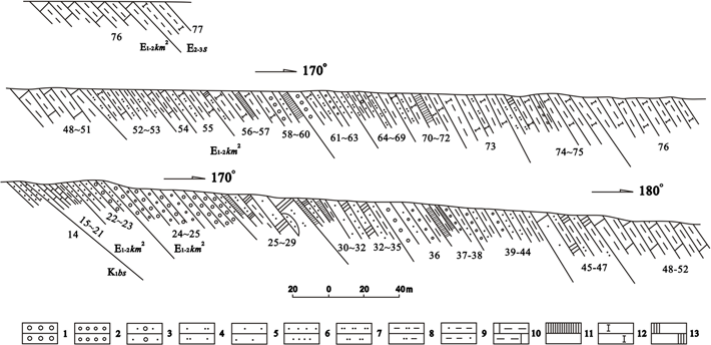
**The profile of the Paleogene Kumugeliemu group in the Kuqa River area.** (After Tarim Geological Team, 1998). 1-medium conglomerate; 2-fine-grained conglomerate; 3-coarse-grained gravel-bearing sandstone; 4-sandstone with unequal particle; 5-coarse-grained sandstone; 6-fine-grained sandstone; 7-siltstone; 8-pelitic siltstone; 9- sandy mudstone; 10- limy mudstone; 11-gypsum rock; 12-lime-bearing rock; 13- gypsiferous rock.

Rocks of the Kumugeliemu Group are distributed along the northern monocline belt between the western Kapushaliang area and the eastern Kezilenuer graben in the Kuqa foreland basin (Tarim geological team (1998). The full sequence of the Kumugeliemu Group is exposed in the Kuqa River profile, which is located in the northeastern part of the Kuqa depression (Figure 2). From base to top, the Kuqa River profile comprises of following lithologies: a gray to light gray marl, a purple–red sandy conglomerate interbedded with mudstone, siltstone and gypsum-bearing evaporite, and gray and red mudstone. Based on these lithological characteristics, the Kumugeliemu Group in the Kuqa River region can be divided into two lithologic associations: (1) a purplish red mudstone with off-white gypsum-bearing rocks and cream-colored mudstone, and (2) a lower part containing grey limestone and gray conglomerate, and an upper part containing purplish red conglomerate, sandstone interbedded with mudstone, and sandstone with gypsum.

### Analytical results and discussion

The results of thermal conductivity analysis are provided in Table 1. The thermal conductivity measurements range from 0.436 to 2.544 W/mK, with an average of 1.380 W/mK. The sandstone and sandy conglomerate yielded thermal conductivity measurements from 1.180 to 2.544 W/mK, with an average of 2.024 W/mK. The gypsum-bearing samples have thermal conductivities of 0.436 to 0.998 W/mK, with an average of 0.744 W/mK. The gypsum-bearing rocks have generally lower thermal conductivity than the sandstone and sandy conglomerate.

**Table 1:** **Results of thermal conductivity of rocks in Kuqa River profile**

Stratum	Spot	Rock type	Thermal conductivity	Thermal diffusivity	Heat capacity
			(W/mK)	(mm^2^/s)	(MJ/m^3^K)
E_1-2km_	1	bottom glutenite	2.034	1.208	1.684
	2	bottom gypsum	0.577	1.314	0.439
	3	bottom gypsum	0.436	0.395	1.103
	4	bottom sandstone	1.180	0.816	1.446
	5	lower sandstone	1.992	0.929	2.144
	6	lower-middle gypsum	0.614	0.527	1.166
	7	lower-middle sandstone	2.544	1.391	1.829
	8	middle sandstone	2.303	1.234	1.866
	9	middle gypsum gravel	0.998	0.587	0.701
	10	middle gypsum	0.964	0.561	1.717
	11	middle including gypsum sandstone	0.873	0.756	1.155
	12	middle-upper sandstone	2.091	1.086	1.925

The thermal conductivities for gypsum obtained here are slightly lower than the values proposed by Kappelmeyer and Haenel (1974) Kappelmeyer pointed out that the highest value of thermal conductivity for gypsum could reach 1.6 W/mK. This discrepancy may reflect the amount of water contained in the samples. For example, Wang and Shi (1989) compared the thermal conductivity of sandstone and mudstone from the Subei Basin under dry and water-saturated conditions, and found that the thermal conductivities were lower under dry conditions. In the present study, the outcropping gypsum rock samples are relatively dry, so the measured values may be low.

We also determined the thermal diffusivity and heat capacity for gypsum-bearing rocks in the Kuqa River profile, which fall in the range from 0.395 to 1.314 mm^2^/s and 0.439 to 1.717 MJ/m^3^K, respectively.

From these results it can be seen that gypsum-bearing rocks have low values of thermal conductivity and thermal diffusivity. Therefore, in addition to being non-porous and non-permeable cap rocks, gypsum-bearing rocks are also good insulators, and are conductive to petroleum generation as well as preservation.

Most previous thermal conductivity studies of representative rocks from the Tarim Basin have focused on outcropping clastic rocks (argillaceous, sandy, or conglomeratic), carbonate rocks, volcanic rocks, or metamorphic rocks (Wang et al. 1999; Feng et al. 2010; Wang et al. 2010; Yu et al. 2010). (Qin 2002) pointed out that the thermal conductivity of rocks from the basin in the northwest of China increased with increasing depth and increasing sample age. Wang et al. (1995a) analyzed the thermal conductivity of more than 220 samples from the Tarim Basin and Kuqa profile, and reported that heat flow values in the Tarim Basin mainly ranged from 40 to 50 mW/m^2^, with the highest values in the middle of the basin, and the lowest values on the western and southwestern edges. The heat flow values obtained from the Kuqa depression were intermediate, ranging from 55 to 56 mW/m^2^.

Many areas of hydrocarbon accumulation have recently been discovered in the Kuqa depression, in regions with closely associated gypsum-bearing rocks (Jia et al. 2002; Wang et al. 2005; Liang et al. 2003; Lin et al. 2002; Zou et al. 2006). Examples are in the Dawan No. 1 Well, Dawan No. 109 Well, Kela No. 2 Well, Dabei No. 1 Well, and Yaha No. 3 Well. Wang et al. (2005) considered that the formation of these oil and gas reservoirs is always connected with gypsum-bearing rocks, as these gypsum-bearing layers are very good cap rocks and are conducive to the preservation of organic matter and to hydrocarbon generation. The strength of gypsum-bearing layers also enables rocks beneath them to maintain high porosity, and to form good hydrocarbon traps by preventing the formation of hydrocarbon pathways. The abnormally high pressure and cracks in the lower parts of gypsum-bearing strata are able to minimize the compaction and diagenesis of subsalt strata, allowing these strata to maintain high porosity (Zhang and Tian 1998). An excellent example is provided by the Cretaceous sandstone below Paleogene gypsum rock in the Qiulitake region, Tarim Basin. This Cretaceous sandstone has very fine fractures, and resultant high porosity, and therefore acts a very good petroleum reservoir.

The 300–1000 m thick Paleogene gypsum-bearing Kumugeliemu Group in the Kuqa depression is the most effective regional cap, and combined with glutenite strata at the reservoir base, constitutes a good reservoir–seal combination. The glutenite strata at the bottom of Kumugeliemu Group are a scour–fill feature, with generally poor sorting and rounding, but with fining-upward normal grading. Above the glutenite, the sandstone package is relatively thin. Medium-to fine-grained sandstones are abundant in the middle–southern part of the group, and have reverse grading. The capping Paleogene gypsum-bearing units (the Kumugeliemu Group) have enabled the basalt glutenite reservoir to maintain a high porosity (Zhu et al. 2009; Shen et al. 2001; Lv and Jin 2000; Song et al. 2011). Based on nearly 1000 test data, from more than 40 wells, the porosity of the Paleogene glutenite reservoir ranges from 3.21% to 28.47%, with an average of 11.5%. Pores are formed by both primary intergranular void spaces and areas of dissolution, while gypsum and calcite are the main cementing minerals. The diagenetic stage of the reservoir is mesogenetic stage A_1_ (Ying et al. 2004; Zhu et al. 2009; Wang et al. 2004). The best reservoir–seal rocks of the Kumugeliemu Group are found mainly in the Dina No. 2 gas field and in the Dongqiu No. 5 well. The Paleogene gypsum-rich layers of the Kuqa depression form an extruding anticlinal trap, beneath which hydrocarbons have accumulated.

## Conclusions

We have measured the thermal conductivity of gypsum-bearing rocks to determine the influence of this rock type on the generation and preservation of oil and gas. The results allow us to draw two main conclusions. Firstly, the thermal conductivity of Paleogene gypsum-bearing rocks from the Kuqa River profile in the Kuqa depression ranges from 0.436 to 0.998 W/mK, with an average of 0.744 W/mK. The thermal diffusivity and heat capacity of these gypsum-bearing rocks range from 0.395 to 1.314 mm^2^/s and from 0.439 to 1.717 MJ/m^3^K, respectively. Secondly, the Paleogene gypsum-bearing rocks from the Kuqa River profile in the Kuqa depression have low thermal conductivity and low thermal diffusivity compared with nearby conglomerate and sandstone units. These gypsum-bearing rocks are therefore good thermal insulators. Combined with the stable nature of the gypsum-bearing rocks, these units make good cap rocks to oil and gas reservoirs under certain conditions.

## Methods

**Figure 3:**
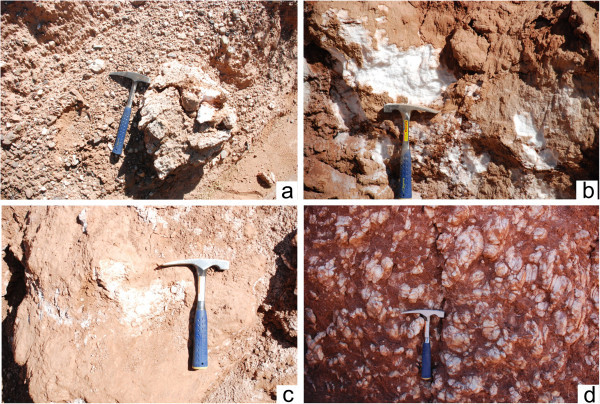
**Field photos of the gypsum rocks of Kumugeliemu group in the Kuqa River area.** **a-**gypsum rock at the bottom, including glutenite; **b-**argillaceous gypsum rock at the bottom; **c-**mud-bearing gypsum rock at the middle-lower part; **d-**gravel-like gypsum rock at the central part.

We investigated the thermal conductivity of gypsum-bearing rocks and nearby conglomerate and sandstone units from the Kuqa River section. Twelve samples were analyzed, spanning from the base to the top of the section. Gypsum color and habit was variable through the sequence, with gypsum at the base of the Kumugeliemu Group being white, massive, and locally with a muddy surface (Figure 3a, 3b); gypsum in the middle–lower part is grey, with mud and loose particles (Figure 3c); and gypsum in the middle of the sequence typically occurs as larger, gravel-sized fragments (Figure 3d).

There are three main techniques for testing thermal conductivity: the hot plate, laser astigmatism (or laser flash), and hot disk methods. Here we have used the hot disk method, as it has generally smaller analytical errors and requires smaller sample volumes compared with the other two techniques. The hot disk method is based on the transient plane source (TPS) technique developed by Dr. Silas Gustafsson (Suleiman et al. 1993; Log and Gustafsson 1995; Gustavsson et al. 1994,1996). In this study, rock thermal conductivity was measured at room temperature and atmospheric pressure, at the Institute of Geomechanics, Chinese Academy of Geological Sciences, Beijing, China.

### Experimental principle

**Figure 4:**
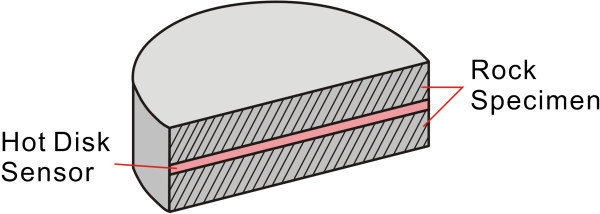
**Sketch map of the sensor and samples during a hot disk measurement.**

We used a Hot Disk Thermal Constants Analyzer system to measure the thermal conductivity, thermal diffusivity, and heat capacity of the rocks. In this instrument, the probe comprises a flat sensor with a continuous double spiral of electrically conducting nickel etched out of thin foil, clad between two layers of Kapton. Although only 0.025 mm thick, the Kapton layers provide electrical insulation between the sample and probe, as well as mechanical strength for the probe. To provide electrical insulation during a hot disk measurement, the sensor is sandwiched between two samples (Figure 4). Different sensor sizes and formats are available to accommodate a large variety of samples. During measurement, a current passes through the nickel and creates a temperature increase. The heat generated then dissipates through the sample, at a rate proportional to the thermal conductivity characteristics of the material. By recording the temperature vs. time response in the sensor, the thermal conductivity characteristics of the material can then be calculated. As the hot disk probe is both the heat source and temperature sensor, the method is very quick and convenient.

The hot disk thermal constant analyzer enables measurements of thermal conductivity over a range from 0.005 to 500 W/mK (with an accuracy of ± 3%), thermal diffusivity from 0.005 to 150 mm^2^/s (accuracy of ± 5%), and volumetric heat capacity from 0.01 to 5 MJ/m^3^K (accuracy of ± 3%). The instrument has a variety of test modules, enabling analysis of several different materials, such as metals, ceramics, or polymers, in block, powder, plaster, film, or anisotropic form. The different probes have radii of 0.49 to 29.4 mm, and have a temperature range of 10 to 1000 K.

### Experimental procedures

- Sample Preparation: All test samples were cut into circular cylinders 50 mm in diameter and 50 mm high. Samples were then polished to be clean and smooth to ensure better contact with the probe.
- Testing: When the Nickel sensor was used, the selected two samples were required. The sensor was then sandwiched between the two halves of the sample material. During measurement, an electric current was passed through the nickel sensor, creating a temperature rise in the surrounding rock. The heat generated dissipated through the sample at a rate determined by the thermal conductivity and other related characteristics of the sample. The thermal conductivity of the measured material was then evaluated by recording the temperature response time. From these data, the thermal diffusivity and heat capacity of the material studied were determined via a fitting function and slope calculation methods.
